# Food insecurity, diet and mental distress among resource insecure students during COVID-19

**DOI:** 10.1093/emph/eoad001

**Published:** 2023-01-11

**Authors:** Miriam C Kopels, Casey J Roulette

**Affiliations:** Department of Anthropology, San Diego State University, San Diego, CA, USA; Department of Anthropology, San Diego State University, San Diego, CA, USA

## Abstract

**Background and objectives:**

It is well documented that college student populations are vulnerable to food insecurity and other adverse environmental conditions. Additionally, exposure to environmental adversity can have deleterious, long-term effects on physical and mental health. This study applies evolutionary life history theory to examine the relationship between environmental adversity, mental distress and diet among resource insecure university students during the COVID-19 pandemic.

**Methodology:**

Structured and semi-structured surveys were used to assess perceptions of environmental adversity (including mortality risk, food insecurity and resource availability; and changes in these factors over the course of COVID-19), mental distress, diet and use of campus support services. Participants included 51 college students recruited through an economic crisis center located at a large public university in southern California.

**Results:**

Most students were experiencing mental distress and food insecurity, and food insecurity and other components of adversity increased during COVID-19. Food insecurity was significantly associated with both perceived extrinsic mortality risk and mental distress, whereas mental distress was significantly associated with reduced dietary quality and caloric intake. Use of two or more campus support resources and/or living with family or rent free disrupted the associations of food insecurity with extrinsic mortality risk and mental distress.

**Conclusion and Implication:**

This study contributes to a growing body of applied evolutionary frameworks concerned with the health and wellbeing of economically vulnerable populations. It also provides novel insights informed by life history theory into interventions and recommendations for improving support services for financially insecure college students.

## Teaser

This study investigates how COVID-19 impacted environmental adversity, psychological distress, and diet for a population of economically vulnerable students. Mental distress and perceptions of mortality risk were associated with food insecurity, while mental distress negatively impacted diet. We identify factors that may help vulnerable students.

## BACKGROUND AND OBJECTIVES

Food insecurity (FI) is a complex biosocial phenomenon that overlaps with but is distinct from related concepts such as food availability, famine and hunger. FI involves both an individual’s access to food as well as supply, utilization and stability of resources. According to the widely accepted World Health Summit definition, FI households lack the economic and physical means to access ‘sufficient, safe and nutritious food to meet their dietary needs and food preferences for a healthy and active life’ [1].

In 2019, prior to the COVID-19 pandemic, approximately 10.5% of households in the United States (USA) experienced FI—a startlingly high number for a wealthy democratic nation. Individuals who identify as an ethnic or racial minority [2], female [3], a sexual minority [4], immigrant [5] or who are disabled [6] suffer comparatively higher rates of FI than the general US population. Among college students, FI rates are about 4–5 times higher than the US national average, with estimates ranging between 12% and 59% of US students experiencing FI [7]. FI rates are typically higher among college students who are minority status [8] and receive financial aid [8]. During the COVID-19 pandemic, significant increases in the rates of FI were observed among many US households [9], but primarily among those who (i) have children, (ii) identify as Black and/or (iii) experienced unemployment and/or the inability to work due to the pandemic [10]. Many US college students also experienced worsening FI during COVID, with one recent study at a large, public institution showing that 59.6% of *n* = 3206 students who experienced changes in food security status became less food secure during the pandemic [11].

While there is no single ‘all-cause’ factor that explains FI, abundant research highlights the primary role that structural factors play in constraining the dietary security of households. In the USA, financial insecurity is strongly associated with FI through numerous pathways. Some of these include: lack of affordable housing [12] and insufficient social safety nets [13]. Among college student populations, food security is constrained by numerous structural factors [14], including rising tuition, housing and living expenses, which—when coupled with low income—often force students to choose between healthy meals or safe housing [15]. Looking at qualitative data, other risk factors emerge, including financial and time constraints (that particularly impact commuter students), as well as insufficient resources to provide enough, high-quality food [16]. Looking specifically at risk factors contributing to college student FI during COVID-19, we see associations with (i) changes to housing situation [17]; (ii) losses to employment [17]; (iii) providing financial support to family [18] and (iv) increased cost of groceries [19]; as well as psychological factors, such as anxiety or COVID-related fears [19].

Among all effected populations, food and financial insecurity are associated with deleterious mental and physical outcomes. These include increased risk of overall poor health among US children [20]; dietary deficiencies linked to childhood asthma [20]; significant limitations to daily activities among seniors [20]; diabetes [21] and diminished mental health [22]. Looking to literature published since the onset of COVID, among college students, FI has recently been associated with: (i) poorer academic outcomes [23]; (ii) poorer psychological wellbeing [24] and (iii) an increase in health-related risk factors, such as poorer nutritional intake [25]. Importantly, the structural causes of FI and these (and other) mental and physical health outcomes are rooted primarily outside of an individual’s control and are ultimately the byproducts of a system that perpetuates economic and health disparities.

Life history theory (LHT) is a middle-range evolutionary theory that provides a useful framework to examine the effects of resource insecurity and adverse life conditions on behavior and health. At its core, LHT deals with how organisms evolved to allocate energy across competing life demands through implementing contextually dependent life history strategies, such as when and how much energy to invest in reproductive effort (e.g. producing offspring) that ultimately comes at the expense of energy that can be invested in somatic domains (e.g. growth or maintenance) [26]. Within the LHT framework, environmental adversity is defined as both the degree of morbidity and mortality in the environment (referred to as environmental harshness) and its spatial-temporal variation (referred to as environmental unpredictability) [27]. Environmental adversity works together with density-dependent effects (i.e. resource scarcity) to impact the life history strategies of organisms [28].

Variation in life history strategies, which are widely characterized along a fast–slow contiuum, are mediated in part by environmental adversity [29]. If individuals live in harsh environments with few resources they are more likely to exhibit faster life history strategies, which prioritize quicker maturation, more abundant and faster reproductive effort, less somatic maintenance, and accelerated senescence [29]. Harshness is also separated into two types of mortality risk: (i) intrinsic mortality, which is the risk of local death potentially buffered by behavior and/or culture, and (ii) extrinsic mortality, which is the risk of death that can not be buffered with behavior strategies [30]. Individuals in resource-poor conditions might display more present-oriented, fast life history strategies due to the coupled effects of high waiting costs (each unit of resource that is reserved for the future is a unit that can not be used today to improve conditions in other important fitness-relevant domains) and—when faced with extrinsic mortality risk—high collection risks (the risk, or likelihood, associated with obtaining a future payoff or resource [31]).

LHT has increasingly been applied to examine health disparities among economically disadvantaged populations. For example, while Pepper and Nettle [32] emphasize the primary role that structural factors play in explaining health disparities along socioeconomic gradients, they also argue that health disparities are further exasperated due to shifts in optimal resource allocation in the face of extrinsic mortality risk. According to their Behavioral Constellation of Deprivation framework, structural factors affect long-term health and wellbeing by limiting economic opportunities as well as increasing exposure to mortality risk, which favors reduced investments in long-term maintenance. Similarly, empirical work by Uggla and Mace [33] draws connections between extrinsic risk and future discounting—showing that (i) lower socioeconomic individuals suffer from higher extrinsic mortality risk, and (ii) exposure to extrinsic mortality risk is positively associated with an increase in ward-level crime. Importantly, both Pepper and Nettle [32] and Uggla and Mace [33] argue that public health efforts are best served by identifying the root structural causes of extrinsic mortality risk, since such causes are often associated with phenotypic strategies that discount long-term health and safety. Recently, there has been growing interest in using a LHT framework to examine the physiological and behavioral effects of FI specifically. Burris *et al.* [34], for example, recently demonstrated that females in food insecure households are 4.4 times more likely to experience earlier age of menache than females in food secure households—a potential example of a faster life history strategy when exposed to resource insecure environments.

Here, we examine college students’ perceptions of environmental adversity and linkages to food security, mental distress and diet. We also explore how perceptions of adversity changed during COVID-19 as well as identify factors associated with environmental adversity, mental distress and dietary patterns. Finally, we explore potential buffering mechanisms that may protect students against environmental harshness and associated deleterious outcomes. While we note that extrinsic mortality risk is hypothesized to generate behavioral responses [see Ref. [33]], in this study we do not test this hypothesis. Instead, the scope of this paper is limited to exploring possible linkages between FI, perceptions of extrinsic mortality risk (PEMR) and other components of adversity, diet and mental distress. Ultimately we hope to identify areas that contribute to PEMR that, theoretically, could be addressed to help improve long-term health and economic wellbeing among US college students. As such, this research attempts to answer the call made by Refs. [32] and [33] to elucidate the environmental components contributing to perceptions of extrinsic mortality in specific cultural-ecological contexts. We also contribute to a growing body of applied evolutionary approaches in the behavioral and medical sciences [e.g. Ref. [34]]—many of which employ a LHT framework. Additionally, this project contributes to our understanding of mental distress within the context of environmental adversity, which is underexplored but potentially important to our understanding of the etiology of mental health [see Ref. [35]]. Ultimately, our goal is to present research that can be used to address underlying ecological constraints and facilitate greater investment in long-term student outcomes, such as educational attainment, financial wellbeing and health.

## Methods

A total of *N* = 51 participants completed an online Qualtrics survey and a semi-structured phone interview in the fall semester of 2020 (September–December). During this time classes were conducted remotely with limited access to dormitory housing. Students were recruited in partnership with the Economic Crisis Response Team (ECRT) at San Diego State University (SDSU), who sent recruitment fliers to students in their email listserv. The ECRT is an on-campus department opened to all SDSU students. They provide resources for food or housing security crises. This includes immediate, short-term resources (such as grocery gift cards, referral to the student food pantry or emergency housing), as well as wrap-around services that connects students to resources designed to facilitate their long-term stability. As part of university pedagogy, instructors are encouraged to include information about ECRT in their syllabi. There is also a food pantry on campus (not directly associated with ECRT) that is typically open 10 AM–4 PM two days a week. However, this service was not available during Fall 2020 due to COVID restrictions. Instead, students were directed to community pantries.

Participants were compensated with a $20 Amazon gift card for each survey completed. Research methods were approved by SDSU’s Institutional Review Board. Note that a total of *n* = 118 participants completed the self-administered Qualtrics survey, which also included data not reported here (i.e. on economic wellbeing effort and drug use). Here we only report the results for the subset of participants who also completed the telephone interview.

### Surveys

An online survey was self-administered on the Qualtrics survey hosting site during the Fall 2020 semester. We asked respondents about sociodemographic characteristics (age, gender, sex, ethnicity, income and medical insurance status) as well as questions about perception of mortality risk, food security and perception of resource availability (see appendix for complete survey).

To measure perceptions of extrinsic mortality risk (PEMR) and intrinsic mortality risk (PIMR), we utilized questions developed by Pepper and Nettle [36], which were based on the ‘Subjective Probability of Living until 75 scale’ designed to untangle extrinsic and intrinsic components of mortality risk. We asked participants:

If you made the maximum effort you could make to look after your health and ensure your safety, what do you think the chances would be that you would live to 75 or more?

This question was asked in two iterations, first ‘these days’ (i.e. during COVID-19) and then ‘prior to the COVID-19 pandemic’, which (although this method is unvalidated) allowed us to approximate changes in perception of mortality risk during COVID. This measure requires further psychometric validation for use with college populations, an area for future research.

FI was assessed with the USDA Household Food Security Survey Module (FSSM) six-time short form. The FSSM was asked twice on the self-administered Qualtrics survey. The first iteration asked students to reflect upon the time since the onset of the COVID-19 pandemic (Spring-Fall 2020), and the second iteration asked them to reflect on the 12-month period prior to the pandemic. Although this method is unvalidated, it allowed us a quick, low-burden approximation of changes to food security status over COVID. Similar methodology was recently utilized by [18].

We developed a question to measure participants’ perception of economic, social and mental health services available to them (perceived resource availability or PRA). Retrospective questions for PIMR, FI and PRA were also developed. The retrospective questions asked participants to reflect upon the period before the COVID pandemic, allowing us to compare how perceptions of adversity changed over the course of the pandemic.

The telephone interview comprised of both structured and open-ended survey items measuring (i) non-clinical psychological distress (Kessler-6); a (ii) 24-hr food recall, which included retrospective diet-related questions and (iii) an open-ended semi-structured assessment of student’s living situation, use of campus support service utilization and ratings of the services’ accessibility and usefulness. Students were also asked to provide feedback and recommendations for improvements for assistance programs on campus. To maintain consistency, all telephone interviews were conducted by the same member of the research team. To score the Kessler-6, we used cutoff points developed by [37], which classify participants into serious (K6 scores ≥13), moderate (≥5 K6 < 13), and low/no (K6 < 5) mental distress.

To assess resource utilization, students were asked about their use of economic, housing, food, and/or academic support services. Participants were then asked about the accessibility and usefulness of the campus resources, and responses were scored from 1 to 3 (1 = not accessible/useful, 2 = moderately and 3 = very accessible/useful). Scores were then averaged across all resources to generate overall usefulness and accessibility scores.

Finally, participants were asked open-ended questions about the following: (i) housing situation (including obligations to pay rent); (ii) types of campus resources regularly used; (iii) assessment of the usefulness and accessibility of resources; (iv) ways SDSU can improve student support; (v) the biggest issues currently facing SDSU students (as of Fall 2020); (vi) factors that may impede access to medical care and (vii) current student status. Answers to some questions were treated as list data, and composite salience scores (*∑*) were calculated by summing all individual salience scores (a statistic accounting for rank and frequency) for an item and then dividing by the number of informants.

### Analysis

Exploratory analyses were performed using bivariate tests of associations and multivariate regression analyses, focusing on changes in adversity from pre-COVID to during COVID and factors associated with mortality risk, mental distress and diet. We then explored factors that potentially buffer resource insecure individuals from FI and mental distress. Specifically, bivariate tests of association included *t*-tests, Wilcoxon matched-pairs signed-rank tests, ANOVA and chi-square tests with Fisher’s exact. Pairwise correlations were used to identify potential variables of interest to include in multivariate regression models. Unless otherwise reported, variables that were significant in the pairwise tests of associations were retained for the regression models with the caveat that, due to the limited sample size, only two independent variables were included in each model. Thus, each outcome variable might have two or more models. We report only those models with the lowest AIC and BIC values and/or those with the highest *R*^2^ values. Data analyses were performed using Stata 17.0 BE for Mac.

## Results

Participant’s ages ranged between 18 years to 26 years and older, with 34% of participants being either 21 (*n* = 9) or 25 (*n* = 24) years of age. About 51% of participants were under 22 years. The majority of participants were female (*n* = 34); one participant whose sex at birth was male identified as non-binary. Income was an ordinal variable, ranging between 1 and 10, with each unit corresponding to $5000 USD. Thus, a mean of 2.4 corresponds to an average income of approximately $12,000/year. Median income was between $0 and 4999/year (*n* = 23), with 90% of participants earning fewer than $20,000/year and two-thirds earning less than $10,000/year. About 43% of the participants were underrepresented minority (Hispanic/LatinX, African American and/or Native American or Indigenous) with the majority of participants identifying as Hispanic/LatinX (*n* = 18), followed by Asian/Pacific Islander (*n* = 11), White (*n* = 7), mixed race (*n* = 6), Middle Eastern (*n* = 5), African American (*n* = 3) and Native American/Indigenous (*n* = 1) (see Table 1 for detailed summary statistics).

**Table 1.** Descriptive statistics for demographic, environmental adversity, food security, mental distress, diet and resource use variables

	Obs	Mean	Std. Dev.	Min	Max
Age	51	21.86	2.51	18	26
Sex	51	0.33	0.48	0	1
Income	51	2.41	1.94	1	10
Underrepresented minority	51	0.43	0.5	0	1
Extrinsic risk_precov	51	19.08	16.2	0	79
Extrinsic risk	51	20.35	16.47	0	57
Intrinsic risk_precov	46	36.74	23.5	0	90
Intrinsic risk	45	36.53	19.35	5	90
Resource availability_precov	51	53.33	26.83	10	100
Resource availability	51	46.27	29.1	0	100
Food insecurity_precov	46	2.74	2.25	0	6
Food insecurity	41	3.37	2.43	0	6
Student	51	0.9	0.3	0	1
Mental distress	51	9.65	4.71	1	21
Calories	51	1692.83	841.3	504.32	4757
Diet quality	51	2.04	0.75	1	3
Diet disrupted by neg emotions	51	0.25	0.44	0	1
Diet disrupted by low finances	51	0.27	0.45	0	1
Pays rent (y/n)	51	0.69	0.47	0	1
No. campus support services used	51	1.78	1.25	0	4
Uses food resources	51	0.43	0.5	0	1
Uses housing resources	51	0.18	0.39	0	1
Uses economic resources	51	0.75	0.44	0	1
Uses academic resources	51	0.31	0.47	0	1
Uses other resources	51	0.1	0.3	0	1
Resource accessibility	46	2.4	0.76	1	3
Resource usefulness	46	2.47	0.74	1	3

On average, participants experienced low food security both prior to and during COVID; they were also experiencing moderate mental distress. Dietary quality was on average moderate, and use of campus support resources were rated as moderately accessible and useful.

### Changes in environmental adversity over the course of COVID

We examined four components of environmental adversity and how the components changed over the course of the COVID pandemic: PEMR, PIMR, PRA and FI. There was no significant difference observed in PEMR (*t* [50] = 0.7, *p* = 0.48) or PIMR (*t*[41] = 1.03, *p* = 0.31) from before to during COVID-19. Wilcoxon matched-pairs signed-ranks tests were used to test for differences in the distribution of PEMR and PEMR pre-COVID and between PIMR and PIMR pre-COVID with no significant differences observed. Despite observing a slight increase in mean FI scores from before to during COVID, the increase was also not significant (*t* [38] = 1.96, *p* = 0.058). However, the distribution of FI categories changed significantly. Prior to COVID, 48% of participants had low food security and 28% had very low food security, but during COVID 31% experienced low food security (a decrease of 17%) and more than 41% experienced very low food security (an increase of 13%). This is a significant change in the distribution of FI categories (*χ*^2^[4] = 37.18, *p* < 0.05), with most participants moving from the ‘low’ to ‘very low’ FI categories. PRA also decreased significantly over the course of COVID, from a mean of 53.3 to 46.3 (*t* [50] = –2.27, *p* < 0.05).

### Perceived extrinsic mortality risk

**Figure 1.**
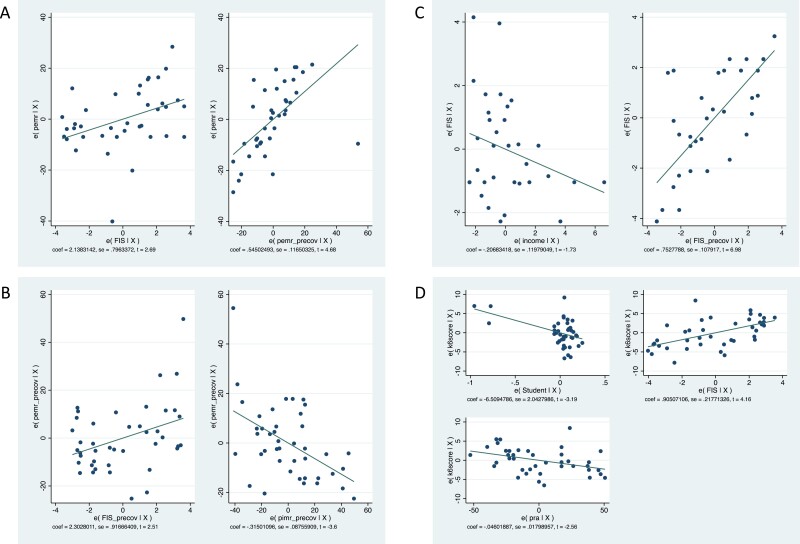
(a) PEMR regressed on FI and PEMR pre-COVID. (b) PEMR pre-COVID regressed on FI pre-COVID and PIMR pre-COVID. (c) FI regressed on income and FI pre-COVID. (d) Mental distress regressed on student status, FI and PRA (Added variable component plots, or partial regression plots, show the relationship between the dependent variable and each independent variable while holding all other variable constant—they show the residual effects of each variable after controlling for the effects of all other variables in the model).

In pairwise correlations, PEMR was significantly associated with PEMR pre-COVID (*r* = 0.685, *p* < 0.05), FI pre-COVID (*r* = 0.466, *p* < 0.05), FI during COVID (*r* = 0.523, *p* < 0.05), and mental distress (*r* = 0.320, *p* < 0.05). We ran several multivariate regression analyses and the model explaining the greatest overall variance in PEMR contained FI and PEMR pre-COVID (Model 1, Table 2 and Fig. 1a) (Model 2, containing FI and FI pre-COVID, had slightly lower AIC and BIC values than model 1, but Model 1 had a much higher R-squared value).

**Table 2.** Comparison of multivariate regression models with perceived extrinsic mortality risk (PEMR) as the dependent variable

	(1)	(2)	(3)	(4)	(5)	(6)
	pemr	pemr	pemr	pemr	pemr	pemr
FI	2.138**	1.553	2.816***			
	(0.796)	(1.492)	(1.009)			
pemr_precov	0.545***			0.552***		0.656***
	(0.117)			(0.122)		(0.107)
FI_precov		2.382		1.976**	2.769***	
		(1.541)		(0.854)	(0.939)	
k6score			0.858		1.09**	0.619*
			(0.556)		(0.449)	(0.367)
_cons	1.939	6.481	1.673	4.355	1.814	1.864
	(3.278)	(3.867)	(5.287)	(3.137)	(4.842)	(4.029)
Observations	41	39	41	46	46	51
*R* ^2^	.539	.3	.316	.469	.312	.499

Standard errors are in parentheses.

*** *p* < .01, ** *p* < .05, * *p* < .1

### Food security

In pairwise correlations, FI had significant associations with mental distress (*r* = 0.45, *p* < 0.05), income (*r* = –0.40, *p* < 0.05), PEMR (*r* = 0.52, *p* < 0.05), PEMR pre-COVID (*r* = 0.37, *p* < 0.05), FI pre-COVID (*r* = 0.78, *p* < 0.05), number of campus resources used (*r* = 0.34, *p* < 0.05) and use of food resources (*r* = 0.41, *p* < 0.05). In this section, we are interested in exploring factors that might explain variation in FI during COVID, such as income, FI pre-COVID and PEMR pre-COVID.

In multivariate regression, the best model of FI-contained income and FI pre-COVID (model 1, Table 3 and Fig. 1c), which together explained 64% of the variance in FI. FI pre-COVID was significant across all models and income was significant at *p* < 0.05 in all models except model 1. PEMR and PEMR pre-COVID were not significant after controlling for FI pre-COVID.

**Table 3.** Multivariate regression model of PEMR pre-COVID regressed on FI pre-COVID and PIMR pre-COVID

	pemr_precov
FI_precov	2.303**
	(0.917)
pimr_precov	–0.315***
	(0.088)
_cons	23.707***
	(4.519)
Observations	42
*R* ^2^	.316

Standard errors are in parentheses.

*** *p* < .01, ** *p* < .05, * *p* < .1.

### Mental distress

In this section, we explore demographic and ecological factors that might contribute to mental distress during COVID (pre-COVID variables are thus excluded from the multivariate regression analyses). In pairwise correlations, mental distress had significant associations with age (*r* = 0.40, *p* < 0.05), PEMR (*r* = 0.32, *p* < 0.05), change in PIMR from before to during COVID (*r* = 0.32, *p* < 0.05), PRA (*r* = -0.45, *p* < 0.05), PRA pre-COVID (*r* = -0.36, *p* < 0.05), FI (*r* = 0.45, *p* < 0.05), current student status (*r* = –0.36, *p* < 0.05) and number of campus resources utilized (*r* = 0.36, *p* < 0.05). We retained age, PRA and student status for the multivariate regression models (preliminary analyses indicated that these variables remained significant after controlling for the effects of all other independent variables.) FI was not significant after controlling for the effects of PEMR and change in PIMR (and vice versa), but when we compared the effects of only these three variables on mental distress, FI consistently outperformed the other two variables. We thus retained FI for the remainder of the multivariate regression analyses. In model comparisons, model 5 (Table 4), which included student status and FI, explained the most variance and had the lowest AIC and BIC scores. However, PRA was significant (at *p* < 0.05) across all models; and model 6, containing only PRA and FI, had the second highest R-squared. Despite concerns given the small sample size, we regressed mental distress on all three variables (student status, FI and PRA) and all three predictors remained significant (not shown); for illustrative purposes, the partial regression effects of mental distress versus student status, FI and PRA are shown in Fig. 1d.

**Table 4.** Comparison of multivariate regression models with food insecurity (FI) as the dependent variable

	(1)	(2)	(3)	(4)	(5)	(6)
	FI	FI	FI	FI	FI	FI
income	–0.207*	–0.449***	–0.395**			
	(.12)	(.165)	(.153)			
FI_precov	0.753***				0.74***	0.799***
	(.108)				(.124)	(.119)
pemr_precov		0.051**		0.005		0.003
		(.02)		(.027)		(.017)
pemr			0.071***	0.075**	0.019	
			(.019)	(.028)	(.018)	
_cons	1.744***	3.586***	3.022***	1.838***	0.89**	1.016**
	(.556)	(.673)	(.656)	(.538)	(.416)	(.424)
Observations	39	41	41	41	39	39
*R* ^2^	.642	.278	.381	.274	.623	.612

Standard errors are in parentheses.

*** *p* < .01, ** *p* < .05, * *p* < .1.

### Dietary quality and caloric intake

In this section, we explore factors related to caloric intake, dietary quality and disruptions to diet. In pairwise correlations, caloric intake was significantly associated with sex (*r* = 0.30, *p* < 0.05) and PRA pre-COVID (*r* = 0.33, *p* = 0.017), and dietary quality was associated with emotional disruptions to diet (*r* = –0.34, *p* < 0.05). ANOVA show that those with lower dietary quality scores are more likely to have serious mental distress (*F* [50] = 6.20, *p* < 0.05) and/or report psychological disruptions to diet (*F* [50] =6.20, *p* < 0.05). (Additionally, when both serious mental distress and psychological disruption to diet are run as presence/absence variables in *t*-tests comparing dietary quality scores, the results are identical.)

Participants reporting financial and/or emotional disruptions to diet during COVID were more likely to have significantly higher perceptions of extrinsic mortality (*t* [49] = –3.06, *p* < 0.05). Additionally, *t*-tests show that individuals who reported psychological disruptions to diet were more likely to have higher psychological distress scores (*t* [49] = –3.53, *p* < 0.05). Finally, significant differences were found in *t*-tests comparing mean caloric intake between those that do and do not report psychological disruptions to diet (1820.7 vs. 1319.0 calories, respectively: *t* [49] = 1.90, *p* < 0.05).

### Factors that potentially disrupt associations among FI, mental distress and PEMR

**Figure 2.**
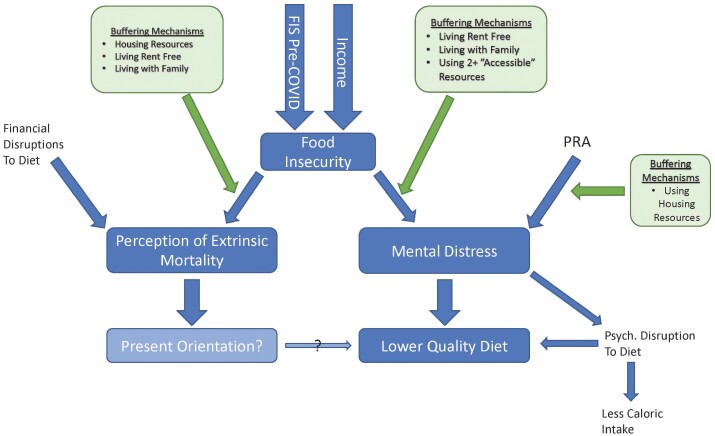
Food insecurity score during COVID-19 was associated with perception of extrinsic mortality risk (PEMR) and mental distress; but PEMR and mental distress were not associated with each other after controlling for food security and perception of resource availability. Mental distress was associated with lower quality diet and psychological disruptions to diet, the latter of which was associated with less caloric intake. The pathways leading from food security to PEMR and mental distress can be buffered through living conditions (not paying rent and/or living with family) and/or use of campus support services, and use of housing resources might protect students who perceive that few resources are available to them from mental distress. Finally, empirical and theoretical research suggests that greater levels of extrinsic mortality risk correlate with a suite of present-oriented behaviors, which could impact diet.

Here, we explore variables that potentially buffer resource insecure individuals from developing FI and/or mental distress. We are interested in how use of campus resources and services, paying rent and/or living with family impacts associations between PEMR and FI, between FI and mental distress and between PRA and mental distress. Results are summarized in Fig. 2.

Among participants who reported not using housing resources (*n* = 34), the association between PEMR and FI was significant (*t* = 3.01, *p* < 0.05), but the association disappeared among participants who reported using housing resources (*n* = 7, *t* = 2.21, *p* = 0.078). Additionally, when participants pay rent, there is a significant association between PEMR and FI (*n* = 29; *t* = 3.45, *p* < 0.05), but the association disappears among participants who do not pay rent (*n* = 12; *t* = 1.48, *p* = .169). Finally, among participants who do not live with family (*p* < 0.05), there is a significant association between PEMR and FI, but the association disappears among participants who live with family (*p* = 0.109). The protective effect of living with family may be due to no or low/informal rent offered in shared family housing situations. In binary logistic regression, students living in shared apartments without family are more likely to report an obligation to pay monthly rent (OR = 28.75, *p* < 0.05).

Among participants who pay rent (*n* = 29), there is a significant association between FI and mental distress (*t* = 3.61, *p* < 0.05), but the association is weak and not significant among participants who do not pay rent (*n* = 12; *t* = 0.52, *p* = 0.612). The association is also significant among participants who report living in a shared apartment (*n* = 22; *t* = 3.02, *p* < 0.05) but disappears among those who report living with family (*n* = 16; *t* = 1.07, *p* = 0.302). It is also worth reporting that participants who live with family were four times more likely to experience a positive change in food security during COVID compared to participants who did not live with family (*n* = 36, *p* < 0.05, OR = 4.12). When two or more campus resources are used, the association between mental distress and FI is no longer significant (*n* = 10; *t* = 0.13, *p* = 0.903). Finally, the association between mental distress and FI was not significant among participants who rated resources as ‘very accessible’ (scores of 3) (*n* = 19; *t* = 1.02, *p* = 0.32) but was significant among participants whose average resource accessibility scores were below three (*n* = 17; *t* = 3.22, *p* < 0.05).

Among participants who reported not using housing resources (*n* = 42), the association between PRA and mental distress was significant (*t* = –4.38, *p* < 0.05), but the association disappeared among participants who reported using housing resources (*n* = 9; *t* = 0.72, *p* = 0.496).

### Implications and conclusions

We discuss our results along four major lines. First, we unpack the association between FI and PEMR and then discuss the relationship between mental distress and environmental adversity. Next, we discuss the effects of environmental adversity and mental distress on diet, and then end with potential buffering mechanisms and recommendations. Overall, our major findings and hypothesized causal arrows are heuristically shown in Fig. 2. It is also important to underscore that the causal factors behind FI and income are likely structural and intersect with issues such as systemic racism and the lack of a supportive social safety net. This fact guides our interpretation of the results. Finally, it is worth noting that FI may have a temporal component, acting as either a chronic or acute condition. It is important to distinguish between these two concepts, as they may have differential causal factors. Exogenous events (such as natural disasters or employment disruptions) are often associated with acute episodes of FI [38], and thus any increases in FI due to COVID-19 may be temporary and of the acute nature. However, our population of students self-identify as resource insecure, and therefore are more likely to have experienced chronic FI over their lifetime [see Ref. [39], association between student FI and childhood FI]. This is something we will explore in follow-up research.

Based on our results and the results of previous empirical and theoretical research, we hypothesize that FI is a key component of perception of extrinsic mortality risk for resource insecure university students. It is well established across empirical studies that food insecure individuals are more likely to experience deleterious health outcomes and greater all-cause mortality risk. Recent work by Ref. [40] suggests that when controlling for demographic and health-risk factors, food insecure individuals have a higher adjusted hazard ratio (HR) for all-cause mortality (HR = 1.46) and cardiovascular-related mortality (HR = 1.75) [40]. Additionally, marginal, moderate and severe food insecurities are all associated with greater likelihood of all-cause premature mortality [41].

Additional research suggests that FI is a structural factor that is extrinsic in nature—meaning that FI individuals have diminished control over the ability to obtain the food required for health and wellbeing. This evidence comes from quantitative data that associates FI with decreased dietary diversity and lower nutritional quality [42, 43] as well as qualitative data that explores the experiences of food insecure households. A study of health knowledge and eating patterns among individuals at risk for FI showed that despite sufficient nutritional knowledge, eating behaviors were often guided by structural factors, such as financial constraints, prohibitively high food prices or poor mental health [44]. Moreover, a qualitative study of food insecure households in Quebec City, Canada showed that frustration with lack of dietary diversity, dietary control and sufficiency were common themes to emerge in interviews, alongside feelings of isolation and psychological distress [45]. Some of these themes were repeated in the current study. In our sample, a quarter of participants reported emotional disruptions to diet, 27% reported financial disruptions to diet, and nearly 14% reported both. This shows that among our population of resource insecure students, there were factors impeding control over dietary choices.

The primary factors associated with mental distress in our study were perception of the availability of campus support services and FI. As such, our findings demonstrate how environmental adversity contributes to the development of mental distress, which is important to broader discussions about the evolution and ecology of mental health [35]. We found no direct effect of perceived mortality risk associated with mental distress once FI, student status and/or resource availability were included in models. This perhaps suggests that resource scarcity, which is primarily a structural factor of campus life, plays a larger role (at least in this population) than mortality risk in the etiology of mental distress.

Mental distress—and not FI or PEMR—had the largest and most significant associations with dietary behaviors, although mental distress itself was associated with FI. Those reporting financial and/or psychological disruptions to diet were more likely to have higher PEMR scores, but the direction of that association is unclear, as extrinsic mortality risk may impact dietary choice in indirect ways, such as through future orientation. Utilizing this concept, Nettle *et al.* [46] put forth an ‘insurance hypothesis’ to provide an evolutionary explanation for the association between FI and obesity observed in Western populations. This hypothesis states that organisms (including humans) in food insecure environments can buffer themselves against periods of famine by increasing caloric consumption and fat storage when resources are readily available. Therefore, it is also possible that perception of extrinsic mortality risk is a factor impacting dietary intake; something that can be explored in future research.

Looking to the structural issues that specifically constrain the dietary choices of resource insecure university students, recent data from Australia shows that food insecure university students are more likely to be dissatisfied with their on-campus food options—showing a deficit of nutritional options completely out of individual control [47]. Additionally, other studies show that food insecure university students are sometimes constrained by feelings of stigma or guilt that prevent them from accessing assistance and expanding their dietary options [48]. In line with these findings, a quarter of participants reported emotional disruptions to diet, 27% reported financial disruptions to diet, and nearly 14% reported both, and the presence of extreme psychological distress and/or psychological disruptions to diet contributed to poor dietary quality and reduced caloric intake.

We identified several factors that buffer the associations between environmental adversity, mental distress and poor diet and that can inform intervention efforts for vulnerable college populations. The association between FI and PEMR is effectively buffered when students live with family, live rent free and/or use housing services on campus. This suggests that structural financial constraints (particularly as they impact housing and food security) are an important part of the pathway linking FI and perception of extrinsic mortality risk (see Fig. 2). The association between FI and psychological distress was effectively buffered under two circumstances: (i) when students were able to live rent free (particularly with family) and (ii) when students utilized two or more campus resources, particularly if those resources were perceived as accessible. Associations between PRA and psychological distress were only buffered if students were able to use housing resources, which shows how important these services are for students living in cities with high housing (and rent) prices.

Targeted methods are a natural outgrowth of evolutionary research that elucidates and addresses sources of perceived extrinsic mortality risk. One of the main goals of this study is to contribute to that research for economically vulnerable university students. We find that FI and financial disruptions to diet are the primary factors contributing to perceived extrinsic mortality risk for high-risk students. This means that feeling out-of-control of diet is likely contributing to perceptions of increased mortality. Evidence suggests that there are several buffering mechanisms available. Therefore, although campus environments are highly variable, we suggest that administrators pay attention to two key pathways to address FI and perceptions of extrinsic risk: Firstly, develop and support programs that actively increase access to food resources on campus. Several recent studies provide potential solutions, including collaborative student-run programs [49]; and increasing knowledge of support services, food literacy and activism [particularly among higher-risk groups like international students and commuters: .16]. Secondly, we recommend strengthening access to housing services, including more well-rounded support for commuters. This is particularly key in high-cost city markets. Housing stability has been shown as a crucial mediator in the link between FI and academic achievement. For example, recent work by [50] makes the convincing argument that the task of ‘hustling’ to find safe, affordable housing contributes to the reproduction of socioeconomic inequalities. Finally, we recommend targeted screenings for psychological distress and FI that can be offered for all economically insecure college students free of charge. All in all, we believe that reducing factors contributing to perceived extrinsic mortality may improve mental health outcomes and contribute to long-term student success and wellbeing.

## Supplementary Material

eoad001_suppl_Supplementary_Appendix

eoad001_suppl_Supplementary_File
