# Supplementary material for: Food insecurity, diet and mental distress among resource insecure students during COVID-19
Source: Evol Med Public Health. 2023 Jan 11;11(1):18–29. doi: 10.1093/emph/eoad001 (PMC9938529; doi:10.1093/emph/eoad001)
Supplement: eoad001_suppl_Supplementary_Appendix [file eoad001_suppl_supplementary_appendix.docx]

# Demographic Characteristics

1. What is your age?
2. What is your gender?
3. What was your sex at birth (female/male)?
4. What is your ethnicity?
5. What is your current gross annual income (about how much money do you earn each year) in $US?
6. Do you currently have medical insurance (no/yes)?

# Perceived Extrinsic and Intrinsic Risk

1. If you made the maximum effort you could make to look after your health and ensure your safety, what do you think the chances would be that you would live to be 75 or more? 0 is ‘no chance’ and 100 is ‘definitely.’
   1. Note for scoring: The extrinsic component of subjective mortality risk (i.e. perceived extrinsic mortality risk [pemr]) is 100 minus this response. It is the portion of perceived mortality risk that the individual believes they cannot reduce via health effort.
2. If you made no effort at all to look after your health and ensure your safety, what do you think the chances would be that you would live to be 75 or more? Again, 0 is ‘no chance’ and 100 is ‘definitely.
   1. Note for scoring: The perceived intrinsic mortality risk (pimr) variable is the difference between the preceding question and this one (i.e. q7 – q8 = pimr) component of subjective mortality risk (i.e. perceived extrinsic mortality risk) is 100 minus this response. It is the portion of perceived mortality risk that the individual believes they cannot reduce via health effort.

# US Household Food Security Survey Module: Six-Item Short Form

Transition into Module:

These next questions are about the food eaten in your household in the last 12 months, since (current month) of last year and whether you were able to afford the food you need.

Now I’m going to read you several statements that people have made about their food situation. For these statements, please tell me whether the statement was often true, sometimes true, or never true for (you/your household) in the last 12 months—that is, since last (name of current month).

a. Note for scoring: See short form 2012 pdf for coding responses.

1. The first statement is, “The food that (I/we) bought just didn’t last, and (I/we) didn’t have money to get more.” Was that often, sometimes, or never true for (you/your household) in the last 12 months?

[ ] Often true

[ ] Sometimes true [ ] Never true

[ ] DK or Refused

1. “(I/we) couldn’t afford to eat balanced meals.” Was that often, sometimes, or never

true for (you/your household) in the last 12 months?

[ ] Often true

[ ] Sometimes true [ ] Never true

[ ] DK or Refused

1. In the last 12 months, since last (name of current month), did (you/you or other adults in your household) ever cut the size of your meals or skip meals because there wasn't enough money for food?

[ ] Yes

[ ] No (Skip AD1a) [ ] DK (Skip AD1a)

1. [IF YES ABOVE, ASK] How often did this happen—almost every month, some months but not every month, or in only 1 or 2 months?

[ ] Almost every month

[ ] Some months but not every month [ ] Only 1 or 2 months

[ ] DK

1. In the last 12 months, did you ever eat less than you felt you should because there wasn't enough money for food?

[ ] Yes

[ ] No

[ ] DK

1. In the last 12 months, were you every hungry but didn't eat because there wasn't enough money for food?

[ ] Yes

[ ] No

[ ] DK

# Perceptions of availability of social, economic, and mental health services

1. Do you feel that the social, economic, and/or mental health services you need for economic stability are sufficiently available and accessible to you? 0 is 'I feel like there are no services that are available or accessible to me' and 100 is 'absolutely all of the services I need are available and accessible to me'.

# Reported Effort in Long-term Economic Maintenance

1. How much effort do you make to look after your economic wellbeing and security these days? 0 is 'no effort at all' and 100 is 'the maximum effort you could make'

# Time Perspective and Planning Ahead Components of Future Orientation Scale

Transition into Module:

These next ten questions (#’s 17-26) contain pairs of statements separated by the word *BUT*. I will read each pair of statements aloud and ask you to choose the statement that best describes you. Once you select the statement, I will then ask you if the statement is *really true* or *sort of true*.

- 1. Note for conducting survey: We are only measuring the Time Perspective and Planning Ahead Components of Future Orientation Scale. These are questions 2,5, 8, 11, 14, and questions 1, 6, 7, 12, 13, respectively. This means that we will not be asking questions 3, 4, 9, 10, or 15.
  2. All items are scored left to right on a scale of 1-4. Reverse score items 1,3,4,6,8,11, and 14. See Steinberg et al. 2009 for more info on methods and scoring.

**Future Orientation Scale**


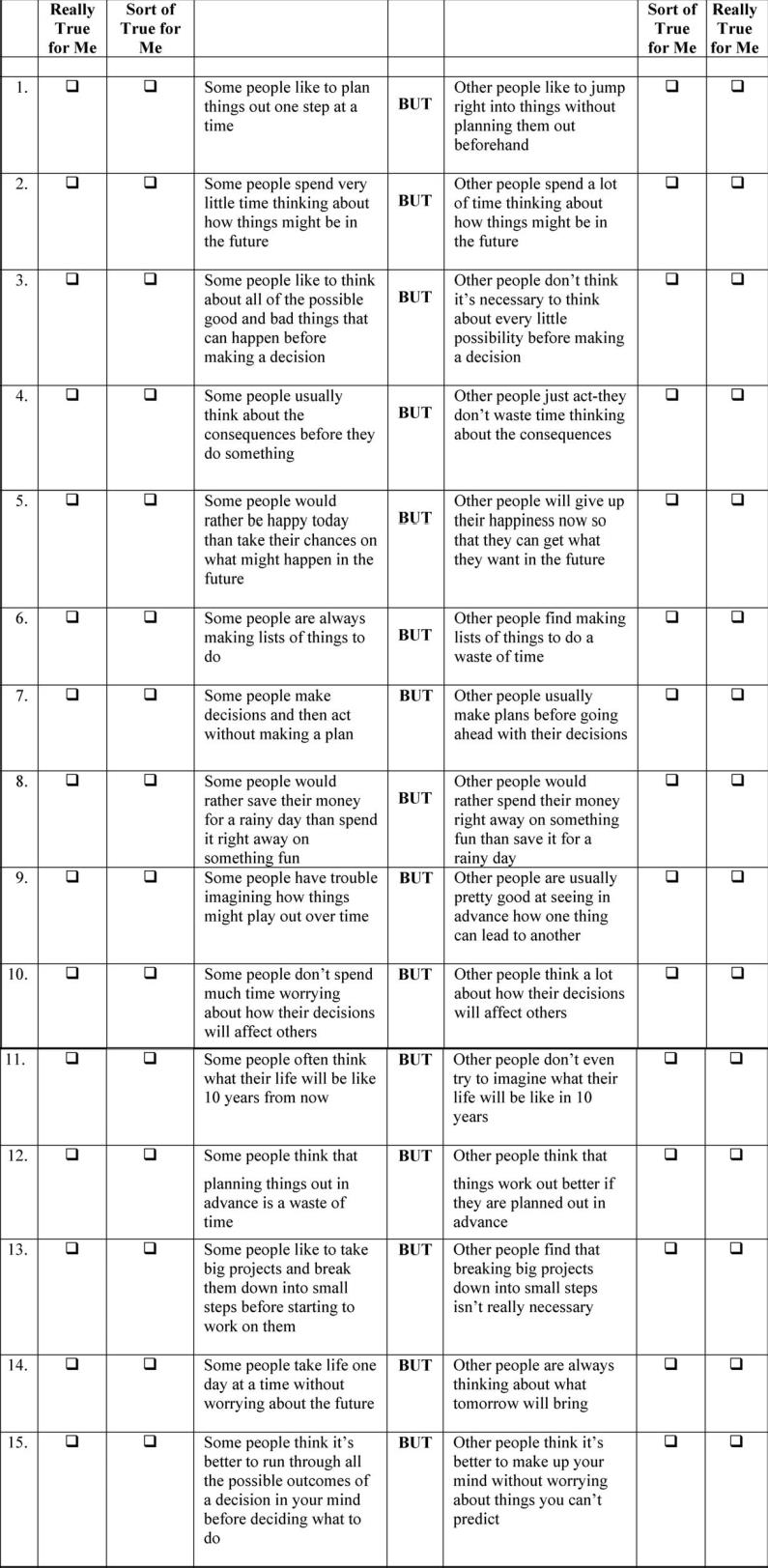


# Kessler-6 Psychological Distress Scale

1. The next questions are about how you have been feeling during the **past 30 days**. About how often during the past 30 days did you feel **nervous** — would you say **all** of the time, **most** of the time, **some** of the time, **a little** of the time, or **none** of the time?
   1. ALL
   2. MOST
   3. SOME
   4. A LITTLE
   5. NONE
2. (IF VOL) DON’T KNOW
3. (IF VOL) REFUSED
4. During the past 30 days, about how often did you feel **hopeless** — **all** of the time, **most**

of the time, **some** of the time, **a little** of the time, or **none** of the time?

- 1. ALL
  2. MOST
  3. SOME
  4. A LITTLE
  5. NONE

1. (IF VOL) DON’T KNOW
2. (IF VOL) REFUSED
3. During the past 30 days, about how often did you feel **restless or fidgety**? (IF NEC: **all**, **most**, **some**, **a little**, or **none** of the time?)
   1. ALL
   2. MOST
   3. SOME
   4. A LITTLE
   5. NONE
4. (IF VOL) DON’T KNOW
5. (IF VOL) REFUSED
6. How often did you feel **so depressed that nothing could cheer you up**? (IF NEC: **all, most**, **some**, **a little**, or **none** of the time?)
   1. ALL
   2. MOST
   3. SOME
   4. A LITTLE
   5. NONE
7. (IF VOL) DON’T KNOW
8. (IF VOL) REFUSED
9. During the past 30 days, about how often did you feel **that everything was an effort**? (IF NEC: **all, most**, **some**, **a little**, or **none** of the time?)
   1. ALL
   2. MOST
   3. SOME
   4. A LITTLE
   5. NONE
10. (IF VOL) DON’T KNOW
11. (IF VOL) REFUSED
12. During the past 30 days, about how often did you feel **worthless**? (IF NEC: **all, most**, **some**, **a little**, or **none** of the time?)
    1. ALL
    2. MOST
    3. SOME
    4. A LITTLE
    5. NONE
13. (IF VOL) DON’T KNOW
14. (IF VOL) REFUSED
15. The last set of questions asked about feelings that might have occurred during the past 30 days. Taking them altogether, did these feelings occur **more often** in the past 30 days than is usual for you, **about the same** as usual, or **less often** than usual?
    1. MORE OFTEN THAN USUAL **GO TO Q33b**
    2. ABOUT THE SAME AS USUAL **GO TO Q34**
    3. LESS OFTEN THAN USUAL **GO TO Q33a**
    4. (IF VOL) NEVER HAVE THESE FEELINGS **GO TO Q34**
16. (IF VOL) DON’T KNOW **GO TO Q34**
17. (IF VOL) REFUSED **GO TO Q34**

33a. **A lot** less than usual, **somewhat** less, or **only a little** less than usual? 1. A LOT **GO TO Q34**

1. SOMEWHAT **GO TO Q34**
2. A LITTLE **GO TO Q34**
3. (IF VOL) DON’T KNOW **GO TO Q34**
4. (IF VOL) REFUSED **GO TO Q34**

33b. **A lot** more than usual, **somewhat** more, or **only a little** more than usual?

1. A LOT **GO TO Q34**
2. SOMEWHAT **GO TO Q34**
3. A LITTLE **GO TO Q34**
4. (IF VOL) DON’T KNOW **GO TO Q34**
5. (IF VOL) REFUSED **GO TO Q34**
6. **A lot** more than usual, **somewhat** more, or **only a little more** than usual?
   1. A LOT
   2. SOMEWHAT
   3. A LITTLE
7. (IF VOL) DON’T KNOW
8. (IF VOL) REFUSED

# INTERVIEWER CHECKPOINT

1. R ANSWERED “A LITLE,” “SOME,” “MOST,” OR “ALL” TO AT LEAST ONE QUESTION IN

THE Q1 SERIES

1. ALL OTHERS **END SECTION**
2. The next questions are about how these feelings may have affected you in the past 30 days. How many days out of the past 30 were you **totally** unable to work or carry out your normal activities because of these feelings?

NUMBER OF DAYS

1. (IF VOL) DON’T KNOW
2. (IF VOL) REFUSED

# INTERVIEWER CHECKPOINT

1. R ANSWERED “30” IN RESPONSE TO Q4 **GO TO Q7**
2. ALL OTHERS
3. [Not counting (that day/those days)], how many days in the past 30 were you able to do only half or less of what you would normally have been able to do because of these feelings?

NUMBER OF DAYS

1. (IF VOL) DON’T KNOW
2. (IF VOL) REFUSED
3. During the past 30 days, how many times did you see a doctor or other health professional about these feelings?

NUMBER OF TIMES

1. (IF VOL) DON’T KNOW
2. (IF VOL) REFUSED
3. During the past 30 days, how often have physical health problems been the main cause of these feelings – **all** of the time, **most** of the time, **some** of the time, **a little** of the time, or **none** of the time?
   1. ALL
   2. MOST
   3. SOME
   4. A LITTLE
   5. NONE
4. (IF VOL) DON’T KNOW
5. (IF VOL) REFUSED

# 24-Hour Food Recall (to include all meals, snacks, and beverages including alcohol and caffeinated drinks)

1. 24 Hour Record

| Time | Quantity Eaten | Details of food and drink |
| --- | --- | --- |
|  |  |  |
|  |  |  |
|  |  |  |
|  |  |  |
|  |  |  |
|  |  |  |
|  |  |  |
|  |  |  |
|  |  |  |
|  |  |  |
|  |  |  |
|  |  |  |
|  |  |  |
|  |  |  |
|  |  |  |
|  |  |  |
|  |  |  |
|  |  |  |

|  |  |  |
| --- | --- | --- |
|  |  |  |
|  |  |  |
|  |  |  |
|  |  |  |
|  |  |  |
|  |  |  |
|  |  |  |
|  |  |  |
|  |  |  |
|  |  |  |

1. Is this a typical day (yes/no) .

40a. If not, give an example of a typical day, if you wish

| Time | Quantity Eaten | Details of food and drink |
| --- | --- | --- |
|  |  |  |
|  |  |  |
|  |  |  |
|  |  |  |
|  |  |  |
|  |  |  |
|  |  |  |
|  |  |  |
|  |  |  |
|  |  |  |
|  |  |  |
|  |  |  |
|  |  |  |
|  |  |  |
|  |  |  |
|  |  |  |

# Self-Reports of Tobacco and Cannabis Consumption

1. Do you use tobacco or nicotine products, including smoking cigarettes, chewing tobacco, snus, and/or vape products (yes/no)? .
2. About many cigarettes/cans of chew/pulls of vape pen do you use on a typical day?

.

42a. A typical Week? .

1. Do you use cannabis or products containing THC, including cannabis flower, edibles, and/or vape products (yes/no)? .
2. About much cannabis or THC containing products do you consume (smoke/eat/inhale) in a typical week? .

44a. A typical month? .
