# Supplementary material for: Food insecurity, diet and mental distress among resource insecure students during COVID-19
Source: Evol Med Public Health. 2023 Jan 11;11(1):18–29. doi: 10.1093/emph/eoad001 (PMC9938529; doi:10.1093/emph/eoad001)
Supplement: eoad001_suppl_Supplementary_File [file eoad001_suppl_supplementary_file.docx]

Supplemental File

The retrospective use of the FSSM to capture FI changes during COVID may have some limitations: 1) it remains to be validated; 2) student populations may overly-generalize when asked to think about food access retrospectively [1]; and 3) when integrated with qualitative data, this method tends to underreport changes in FI at the most extreme end of the distribution, something we will unpack in future papers. However, this method was used across several studies following the onset of COVID-19, and was able to provide low-burden, fast approximations of changes to FI as the pandemic unfolded – further research is required to address limitations [see: 2–4].

During the 24-hour food recall, the past 24-hour period was broken into chunks, and participants were asked what they ate and drank in the morning, afternoon, evening, and night hours. Questions were asked about preparation and ingredients used during cooking, and serving sizes were estimated by relating the size of a food item to a typical household object (i.e., a spoon) as well as hand measurements (i.e., a “thumb size” serving of cheese, or a “palm size” serving of chicken). Although this method has potential sources of error when it comes to estimating dietary variation and how habits change of time [5], because of the semi-structured method, there is greater opportunity for detailed descriptions of food quantities and preparations, and there is greater flexibility when it comes to the types of analyses that can be done with 24-hour recall data [6].

Caloric intake was then calculated by converting the 24-hour dietary recall into caloric estimates using the USDA Food Central Database [7]. Nutritional quality was calculated by converting the dietary recall into an estimate of quality using guidelines from the USDA Healthy Eating Index (HEI) to group foods and beverages into low, moderate, and high-quality categories (scored 1-3). We defined “low quality” items as those recommended to make up <10% of the diet (i.e., added sugars, saturated fats, alcohol), “high quality” items are those included in the HEI healthy eating pattern (i.e., fruits, vegetables, fat-free dairy), and “moderate quality” items were foods that include both low- and high-quality ingredients (i.e., a sandwich with whole grains, vegetables, high-fat cheese, and high-sodium processed meats). Scores for each item were then averaged to yield an overall dietary quality score for each participant. This method allowed us to quickly assess dietary quality using established guidelines. However, because we did not score and weigh the data using dietic methodology we are unable to identify specific nutritional deficiencies or make recommendations for nutritional interventions for this population. Future research will include analyses that generate HEI scores using established methodologies [see: 8].

1. Nikolaus CJ, Ellison B, Nickols-Richardson SM. College students’ interpretations of food security questions: results from cognitive interviews. *BMC Public Health* 2019;**19**:1282.

2. Soldavini J, Andrew H, Berner M. Characteristics associated with changes in food security status among college students during the COVID-19 pandemic. *Transl Behav Med* 2021;**11**:295–304.

3. Adams EL, Caccavale LJ, Smith D *et al.* Food Insecurity, the Home Food Environment, and Parent Feeding Practices in the Era of COVID-19. *Obesity* 2020;**28**:2056–63.

4. Niles MT, Bertmann F, Belarmino EH *et al.* The Early Food Insecurity Impacts of COVID-19. *Nutrients* 2020;**12**:2096.

5. Dodd KW, Guenther PM, Freedman LS *et al.* Statistical Methods for Estimating Usual Intake of Nutrients and Foods: A Review of the Theory. *J Am Diet Assoc* 2006;**106**:1640–50.

6. Baranowski T. 24-hour recall and diet record methods. *Nutr Epidemiol* 2012;**40**:49–69.

7. FoodData Central. 2021.

8. Krebs-Smith SM, Pannucci TE, Subar AF *et al.* Update of the Healthy Eating Index: HEI-2015. *J Acad Nutr Diet* 2018;**118**:1591–602.
